# Perdeuterated GbpA Enables Neutron Scattering Experiments of a Lytic Polysaccharide Monooxygenase

**DOI:** 10.1021/acsomega.3c02168

**Published:** 2023-07-31

**Authors:** H. V. Sørensen, Mateu Montserrat-Canals, Jennifer S. M. Loose, S. Zoë Fisher, Martine Moulin, Matthew P. Blakeley, Gabriele Cordara, Kaare Bjerregaard-Andersen, Ute Krengel

**Affiliations:** † Department of Chemistry, University of Oslo, NO-0315 Oslo, Norway; ‡ Centre for Molecular Medicine Norway, University of Oslo, NO-0318 Oslo, Norway; § Faculty of Chemistry, Biotechnology and Food Science, Norwegian University of Life Sciences (NMBU), NO-1340 Ås, Norway; ∥ Science Directorate, European Spallation Source ERIC, P.O. Box 176, SE-221 00 Lund, Sweden; ⊥ Department of Biology, Lund University, 35 Sölvegatan, SE-223 62 Lund, Sweden; # Life Sciences Group, Institut Laue-Langevin, 71 avenue des Martyrs, 38042 Cedex 9 Grenoble, France; ∇ Large-Scale Structures Group, Institut Laue-Langevin, 71 avenue des Martyrs, 38042 Grenoble, France

## Abstract

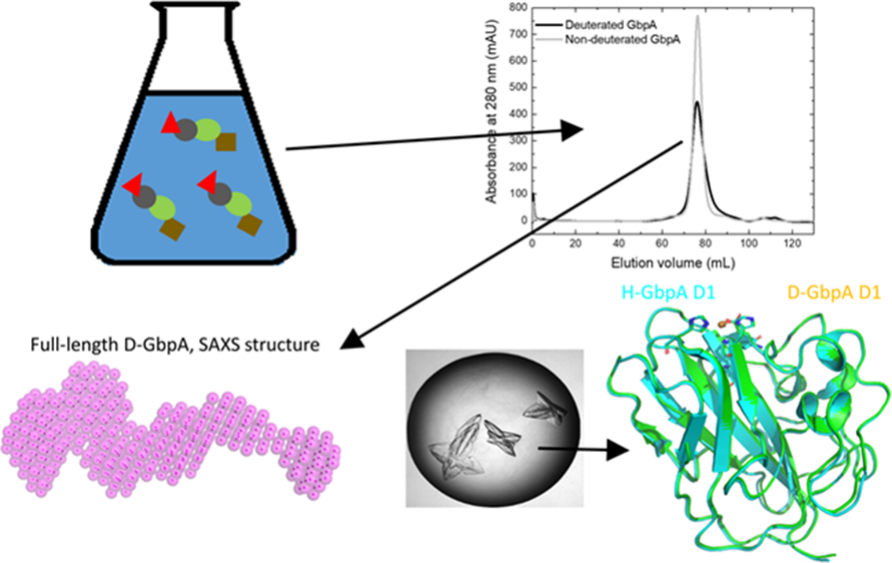

Lytic polysaccharide monooxygenases (LPMOs) are surface-active redox enzymes that catalyze the degradation of recalcitrant polysaccharides, making them important tools for energy production from renewable sources. In addition, LPMOs are important virulence factors for fungi, bacteria, and viruses. However, many knowledge gaps still exist regarding their catalytic mechanism and interaction with their insoluble, crystalline substrates. Moreover, conventional structural biology techniques, such as X-ray crystallography, usually do not reveal the protonation state of catalytically important residues. In contrast, neutron crystallography is highly suited to obtain this information, albeit with significant sample volume requirements and challenges associated with hydrogen’s large incoherent scattering signal. We set out to demonstrate the feasibility of neutron-based techniques for LPMOs using *N*-acetylglucosamine-binding protein A (GbpA) from *Vibrio cholerae* as a target. GbpA is a multifunctional protein that is secreted by the bacteria to colonize and degrade chitin. We developed an efficient deuteration protocol, which yields >10 mg of pure 97% deuterated protein per liter expression media, which was scaled up further at international facilities. The deuterated protein retains its catalytic activity and structure, as demonstrated by small-angle X-ray and neutron scattering studies of full-length GbpA and X-ray crystal structures of its LPMO domain (to 1.1 Å resolution), setting the stage for neutron scattering experiments with its substrate chitin.

## Introduction

Lytic polysaccharide monooxygenases (LPMOs) are surface-active enzymes that introduce breaks in chitin, cellulose, or other polysaccharide layers through a copper-dependent redox reaction. Conserved among all active LPMOs is the histidine-brace motif, where a copper-ion is coordinated by the amino group and a side-chain nitrogen of the N-terminal histidine together with a side-chain nitrogen of a second histidine residue.^1−3^ The enzymatic reaction is initiated by the reduction of Cu(II) to Cu(I) by an electron donor and subsequent activation of an oxygen co-substrate. It is unclear if the oxygen co-substrate is O_2_ or H_2_O_2_, with the growing consensus being that H_2_O_2_ is the more likely co-substrate.^4−6^ The natural electron donor likely varies significantly between organisms.^7^

Interestingly, the function of LPMOs often goes beyond polysaccharide conversion. Several LPMOs are part of multi-domain proteins, often with an additional carbohydrate-binding module. Some LPMOs have shown to be key virulence factors for pathogenic bacteria, with importance for colonization. One example is the AA10 class LPMO, *N*-acetylglucosamine-binding protein A (GbpA) of *Vibrio cholerae*. GbpA is a four-domain protein with chitin affinity of the first and fourth domains, and LPMO activity in the first domain.^8,9^ This domain in addition exhibits affinity for human intestinal mucin,^8,10^ a feature likely important for the colonization of the small intestine by *V. cholerae*.

While the structures of several LPMOs have been determined to atomic resolution by X-ray crystallography, including the first three domains of GbpA,^8^ and all four domains of the close homologue *Vh*LPMO10A from *Vibrio campbellii*,^11^ the functional information has been limited, in part due to the inability to map hydrogen atoms in the structures. For redox-active enzymes like LPMOs, neutron macromolecular crystallography (NMX) is a strong complementary method to X-ray crystallography that can reveal additional information.^12^ With NMX, functionally relevant hydrogens can be modeled even at relatively low (∼2.5 Å) resolution.^13^ Furthermore, neutrons are non-ionizing,^13^ leaving the active site of LPMOs intact throughout the diffraction experiment. Unfortunately, neutron sources have a very low flux, and hydrogen atoms have a high incoherent scattering; this diminishes the signal-to-noise ratio for the crystallographic data. Longer data collection times (e.g., multiple days) and large crystal volumes (0.1–1.0 mm^3^) can therefore be required to determine a neutron protein crystal structure. Also, hydrogen has a negative coherent scattering length compared to the positive scattering lengths of carbon, nitrogen, sulfur, and oxygen, which may cause density cancellation effects in neutron maps at intermediate resolution.^13^ These issues can be resolved with protein deuteration. Deuterium has strong positive coherent scattering and incoherent scattering that is essentially negligible compared to that of hydrogen.^13^ This alleviates the sample volume requirement by as much as a factor of ten. A few neutron crystal structures of LPMOs have already been determined;^14−17^ however, none of them is perdeuterated, enabling mapping of only select hydrogen atoms.

Other neutron techniques that can benefit from protein deuteration are small-angle neutron scattering (SANS) and neutron reflectometry (NR), where contrast-matching can help visualize individual components in a complex, provided that the components have sufficiently different scattering-length densities (SLD). Deuterated biomolecules have very different SLDs from non-deuterated biomolecules. Using SANS or NR on deuterated LPMOs can therefore yield information on the interactions with other biomolecules, most obviously the polysaccharide substrates. An extensive review of deuteration methodologies/protocols/applications for neutron scattering has been given by Haertlein et al.^18^

Perdeuteration of proteins by expression in deuterated bacterial cultures can be a challenging and time-consuming process. Protocols usually require adaptation of the cells to increasing amounts of D_2_O; however, Cai et al.^19^ recently developed a faster and simpler protocol, yielding high amounts of perdeuterated proteins. We adapted this protocol for both full-length (FL) GbpA (GbpA-FL) and for its first domain (GbpA-D1), scaled up production at international deuteration facilities, and characterized perdeuterated GbpA-FL (D-GbpA-FL) by small-angle X-ray scattering (SAXS) and SANS. The protein retained its catalytic activity. We also crystallized and determined the structure of deuterated GbpA-D1 (D-GbpA-D1) using X-ray crystallography and compared it with the X-ray structure of the hydrogenated protein (H-GbpA-D1).

These results demonstrate the feasibility of perdeuterating LPMOs for neutron-based structural biology studies, with the promise of increased knowledge for the functional mechanisms of this important class of enzymes.

## Materials and Methods

### Materials

Glycerol-d_8_ (99% D) and deuterium oxide (99.9% D) were bought from ChemSupport AS (Hommelvik, Norway). For the experiments at D-lab and DEMAX, these chemicals were purchased from Eurisotop and Sigma-Aldrich, respectively. K_2_HPO_4_, KH_2_PO_4_, Na_2_HPO_4_, NH_4_Cl, and glycerol were from VWR (Oslo, Norway). All other chemicals and chemical competent cells were purchased from Sigma-Aldrich (Merck Life Science AS, Oslo, Norway).

### Production of Hydrogenated GbpA (H-GbpA)

Unless specified otherwise, all the work described in this study was carried out with expression constructs provided by the Vaaje-Kolstad laboratory (Norwegian University of Life Sciences). They are described in detail by Wong et al.^8^ Briefly, the GbpA-FL sequence was cloned from the genomic DNA of *V. cholerae* strain N1RB3 into a pET-22b vector between the *NdeI* and *XhoI* sites. The construct for expressing GbpA-D1 was obtained by the addition of two codon stops at position 203. The natural tag for protein secretion (amino acids 1–23) is cleaved off by the *E. coli* expression system. The pET-22b vector contains an ampicillin resistance gene, exploited for selection in growth and expression phases.

H-GbpA-FL was expressed in BL21(DE3) STAR cells transfected with the GbpA-encoding plasmid using Terrific Broth (TB), Luria Bertani (LB), or non-deuterated M9glyc+ media (Table 1, but with non-deuterated glycerol). For expression in TB or LB media, cells were first grown in 50 mL pre-cultures on INFORS culture shakers at 37 °C and 130 rpm to an optical density at 600 nm (OD_600_) of 6–8 absorption units (AU), then transferred to 1 L cultures, and grown at 37 °C to an OD_600_ of 0.8 before induction with isopropyl β-d-1-thiogalactopyranoside (IPTG) at a final concentration of 1 mM. The temperature was lowered to 20 °C after induction, and H-GbpA-FL was expressed for 18 h.

**Table 1:** Composition of Deuterated Media (M9+ Compared to M9glyc+ and ModC1; Ingredients for 1 L Media)

M9+ medium^19^		M9glyc+ medium (this work)	
K_2_HPO_4_	19.0 g	K_2_HPO_4_	19.0 g
KH_2_PO_4_	5.0 g	KH_2_PO_4_	5.0 g
Na_2_HPO_4_	9.0 g	Na_2_HPO_4_	9.0 g
K_2_SO_4_	2.4 g	K_2_SO_4_	2.4 g
d-Glucose-d_7_	18.0 g	Glycerol-d_8_	18.0 g
NH_4_Cl	5.0 g	NH_4_Cl	5.0 g
Trace element solution^a^	1.0 mL	Trace element solution^a^	1.0 mL
MEM	10.0 mL	MEM^b^	10.0 mL
MgCl_2_	0.95 g	MgCl_2_	0.95 g^c^
			

ModC1 medium^20,21^	
NH_4_Cl	2.58 g
KH_2_PO_4_	2.54 g
Na_2_HPO_4_	4.16 g
K_2_SO_4_	1.94 g
Glycerol-d_8_	2 g
d-algal extract	10 mL
Trace element solution^d^	1.0 mL
Vitamin mix^e^	1.0 mL
MgSO_4_(7H_2_O)	0.67 g
FeSO_4_(7H_2_O)	20 mg
Trisodium citrate	88 mg

a The trace element solution was made by dissolving the following ingredients in 100 mL H_2_O: 0.6 g FeSO_4_ (7H_2_O), 0.6 g CaCl_2_ (2H_2_O), 0.12 g MnCl_2_ (4H_2_O), 0.08 g CoCl_2_ (6H_2_O), 0.07 g ZnSO_4_ (7H_2_O), 0.03 g CuCl_2_ (2H_2_O), 2 mg H_3_BO_4_, 0.025 g (NH_4_)_6_Mo_7_O_24_ (4H_2_O), 0.5 g EDTA.

b MEM vitamin solution from Sigma-Aldrich.

c MgCl was dissolved in 10 mL D_2_O prior to addition, which prevented precipitation.

d The trace element solution was made by dissolving the following ingredients in 1 L of D_2_O to prepare a 1000× stock solution: 5.1 g MnSO_4_ (H_2_O), 8.6 g ZnSO_4_ (7H_2_O), 0.75 g CuSO_4_ (5H_2_O).

e The vitamin mix solution was made by dissolving the following ingredients in 1 L of D_2_O to prepare a 1000× stock solution: 25 mg biotin, 135 mg vitamin B12, 335 mg thiamine.

**Figure 1:**
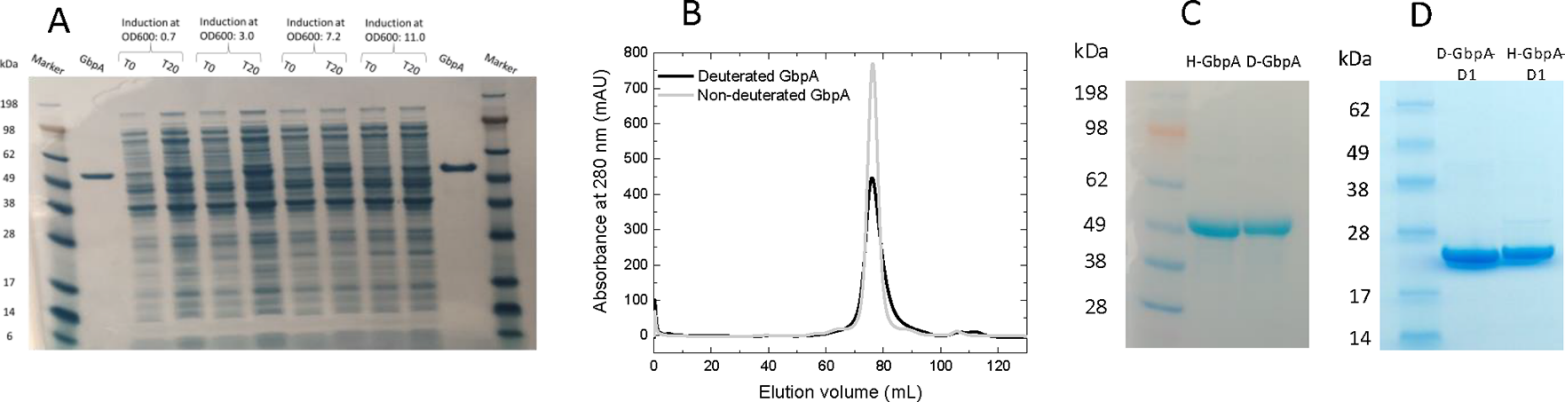
Expression of GbpA in deuterated M9glyc+ medium. (A) SDS-PAGE analysis of full cell proteome, pre-induction (T0), and post-induction (T20), compared for the four different induction points. Seeblue Plus2 marker and purified full-length GbpA (FL) are included for comparison. (B) SEC elution profiles for H-GbpA-FL and D-GbpA-FL (both produced at UiO). Similar elution peaks at the same retention volumes indicate highly comparable hydrodynamic radii. (C, D) SDS-PAGE of H-GbpA-FL and D-GbpA-FL (C) as well as LMPO domain GbpA-D1 (produced at UiO) (D) after SEC.

**Figure 2:**
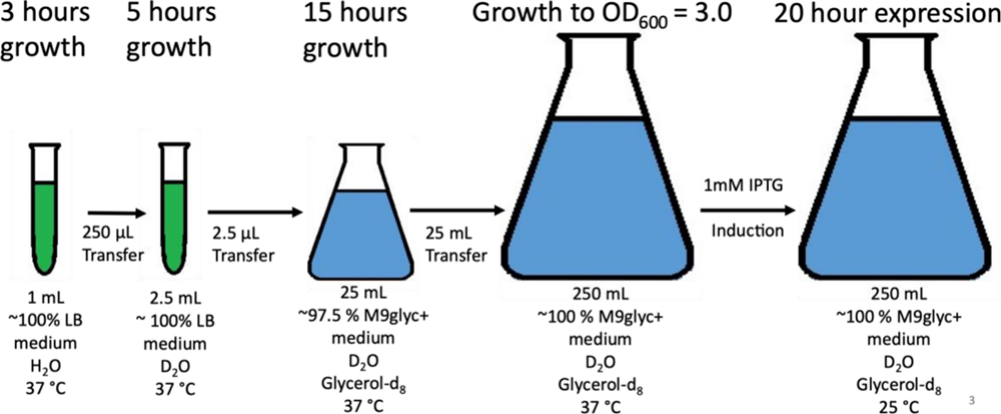
Optimized GbpA deuteration protocol. *E. coli* BL21 Star cells are initially grown in LB medium for 3 h and then transferred to LB medium containing D_2_O, in which the cells are grown for 5 h. Cells are then added to a small M9glyc+ D_2_O pre-culture, in which they are allowed to grow for 15 h until they are transferred to a larger culture. When OD_600_ reaches 3.0, expression is induced by addition of IPTG, and the temperature is lowered from 37 to 25 °C.

For expression of hydrogenated protein (H-GbpA-FL and H-GbpA-D1) in non-deuterated M9glyc+ medium, the cells were grown for 8 h at 37 °C (INFORS shaker, 130 rpm) in 2.5 mL LB/H_2_O medium in 15 mL tubes. This culture was transferred to 25 mL non-deuterated M9glyc+ medium in a 250 mL baffled flask and incubated for another 15 h. Subsequently, we added the culture to 225 mL M9glyc+ media in a 2 L baffled flask. We tested induction with 1 mM IPTG at four different OD_600_ levels (0.7, 3.0, 7.2 and 11.0; Figure S1B) and obtained the best yield at OD_600_ = 3.0 (Figure 1A). After addition of IPTG, the temperature was lowered to 25 °C for 20 h of expression. For a scheme of the protocol, see Figure 2. 100 μM sodium ampicillin was used for selection.

### Production of Deuterated Protein (D-GbpA)

When producing deuterated protein (D-GbpA-FL and D-GbpA-D1), we used the same conditions as for H-GbpA production in minimal media, except for one additional step: After growing BL21(DE3) Star cells containing GbpA-encoding plasmid for 3 h in 1 mL LB/H_2_O medium in 15 mL tubes, this culture was diluted ten times into 2.5 mL LB/D_2_O medium in 15 mL tubes, before incubation for another 5 h. Subsequently, 2.5 mL of this culture were transferred to 25 mL deuterated M9glyc+ medium (Table 1) in a 250 mL baffled flask. This culture was incubated for 15 h and subsequently added to 225 mL deuterated M9glyc+ medium in a 2 L baffled flask, where it remained until OD_600_ reached 3.0 at 37 °C. The production of GbpA was induced by addition of IPTG to a concentration of 1 mM. The culture was left for expression for 20 h at 25 °C. A final concentration of 100 μM sodium ampicillin was used for selection. The cultures remained on culture shakers (INFORS Multitron Standard) at 130 rpm throughout the growth and expression phases. Since we only obtained insufficient yields of D-GbpA-D1 for NMX (∼3 mg/L of media), we contacted international facilities to help us scale up production.

For the production of D-GbpA-D1 at D-lab (ILL) and DEMAX (ESS), a different construct was used (but coding for the same amino acid sequence; UniProt ID: Q9KLD5, residues 24–485). The gbpa gene was codon-optimized and cloned into pET vector pET-26b by GenScript (Leiden, The Netherlands) using the restriction sites *Nco*I and *Xho*I. The pelB leader sequence is cleaved off during post-translational translocation to the periplasmic space to instate the catalytically important His24 as N-terminal amino acid. The C-terminal His-tag was omitted by inclusion of a stop codon at the end of the insert. The pET-26b vector contains a kanamycin resistance gene, which we exploited for selection in growth and expression phases.

For D-GbpA-D1 production at the D-lab deuteration facility of Institut Laue-Langevin (ILL, Grenoble, France; proposal number DL-03-223), the protein was over-produced in *E. coli* strain BL21 (DE3) adapted to growth in deuterated minimal medium,^18^ using the expression construct cloned in the pET-26b plasmid. A 1.9 L (final volume) deuterated high-cell-density fed-batch fermenter culture was grown at 30 °C. Feeding with glycerol-d_8_ was started at an OD_600_ value of about 5. Expression of D-GbpA-D1 was induced at an OD_600_ of about 13 by addition of 1 mM IPTG (final concentration). Cells were harvested at OD_600_ = 15.8, yielding 40 g perdeuterated cell paste (wet weight), thus approximately 20 g/L media. The cell paste was flash frozen and stored at −80 °C to prevent proteolysis before transport to Oslo for further processing.

D-GbpA D1 production in the DEMAX biodeuteration labs of the European Spallation Source (ESS, Lund, Sweden; proposal number 890320) followed a different protocol, without adaptation, using an approach described in Koruza et al.^21^ D-GbpA D1 was expressed in BL21 (DE3) Tuner cells after transformation with GbpA-D1-encoding plasmid using selective growth conditions (i.e., in the presence of kanamycin) on LB agar plates. A 5 mL LB starter culture was grown with kanamycin (50 μg/mL final concentration) after inoculation from a single colony, and was left to grow while shaking at 180 rpm at 37 °C overnight. From this overnight culture, small-scale expression tests were performed in 50 mL LB cultures and a glycerol stock was prepared. For scaling up the yield, the cells were pre-grown in LB medium, a 100 mL overnight culture was started from the glycerol stock, and kanamycin was added (50 μg/mL final concentration). The next morning, 6 × 1 L cultures were inoculated with 10 mL of the overnight culture and fresh antibiotic was added. The cultures were grown in Tunair baffled flasks in a 37 °C incubator while shaking at 200 rpm. When OD_600_ reached 2, the cells were harvested by centrifugation at 5000 × *g* for in a JLA8.100 rotor for 10 min. The cells were gently resuspended in deuterated ModC1 (Table 1) medium and transferred to 6 × 1 L deuterated ModC1 medium in fresh flasks. These media were prepared according to the protocol reported by Koruza et al.^21^ with the addition of 10 mL of *Botryococcus braunii* deuterated algal extract and a reduction in the amount of glycerol-d_8_ to 2 g/L of media. At this point, fresh antibiotic was added, and the cells were allowed to recover for 1 h at 25 °C while shaking at 100 rpm. Thereafter, the shaking was increased to 200 rpm, 1 mM IPTG (final concentration) was added, and expression was left to continue overnight for up to 20 h. Finally, the cells were harvested by centrifugation at 10,000 × *g* for 15 min in a JLA8.1000 rotor and immediately further processed for periplasmic protein extraction as described below. Using this approach, it was possible to obtain 60 g of wet cell paste (i.e., 10 g/L of media) for further processing. The Certificate of Analysis of the material provided is deposited with DOI 10.5281/zenodo.6631673. The periplasmic fraction was frozen at −20 °C and shipped on ice packs for further purification.

### Autolysis Procedure for Deuterated Algal Extract

The procedure for algal autolysis was adjusted and modified from literature descriptions.^22,23^ Microalgae *B. braunii* (UTEX Showa strain, Culture Collection of Algae at the University of Texas at Austin) were continuously cultivated in perdeuterated modified Bolds 3 N medium.^24^ Cells were grown in a 12 h:12 h light–dark cycle and illuminated with 240 μmol photons/m^2^s LED lights in a Multitron Pro incubator (INFORS HT). Cultures were agitated by shaking at 60 rpm, and the atmosphere was kept at 2% CO_2_. The cells were periodically harvested, typically every 14–21 days, by centrifugation in a JLA8.100 rotor (Beckman) at 5000 × *g* for 15 min. The pelleted cells were frozen at −80 °C until further processing. To prepare the autolysate from frozen cells, 400 mL of 99.9% D_2_O was added to 100 g of frozen wet microalgae cells. The cells were thawed and resuspended to a uniform suspension and then incubated at 50 °C in a water bath for 24 h. The digested cell product was centrifuged in a JA-21 rotor (Beckman) at 10,000 × *g* for 20 min. The supernatant was aliquoted in 10 mL tubes and frozen at −20 °C until needed. For modified ModC1 medium preparation, 10 mL of d-algal extract was used per liter of culture media.

### Periplasmic Lysis and Protein Purification

GbpA (both FL and D1, deuterated or hydrogenated) was harvested from the *E. coli* periplasm using the following periplasmic lysis protocol: First, the cell culture was pelleted by centrifugation (10,000 × *g*). The pellet was resuspended in a solution containing 25% sucrose, 20 mM tris(hydroxymethyl)aminomethane (Tris)-HCl pH 8.0 and 5 mM ethylenediaminetetraacetic acid (EDTA) (4–5 mL/g cells), and incubated for 30 min. Thereafter, the cells were once again pelleted and resuspended in ∼50 mL (4–5 mL/g cells) solution of 5 mM MgCl_2_, 1 mM phenylmethylsulfonyl fluoride (PMSF) and 0.25 mg/mL lysozyme. The suspension was incubated for 30 min on ice, pelleted, and the GbpA-containing supernatant was subjected to purification.

GbpA (both FL and D1, deuterated or hydrogenated) was purified by anion-exchange and size-exclusion chromatography. Anion-exchange chromatography (AEX) was performed using a HiTrap Q HP 5 mL column (Cytiva) connected to an ÄKTA Start protein purification system (GE Healthcare). After loading, the protein was eluted by a salt gradient from 100 to 400 mM NaCl buffered with 20 mM Tris–HCl pH 8.0. Size-exclusion chromatography (SEC) was performed using a HiLoad Superdex 200 prep grade column on an ÄKTA Purifier system (GE Healthcare) in a running buffer containing 20 mM Tris–HCl pH 8.0 and 100 mM NaCl. For GbpA-D1, we used a salt gradient for elution during AEX from 50 to 400 mM and performed SEC using a Superdex 75 Increase 10/300 column (Cytiva) on an ÄKTA pure system (GE Healthcare).

### Determination of Deuterium Content

To determine the deuteration level, deuterated and non-deuterated GbpA-FL were dialyzed into MilliQ-purified H_2_O and measured by MALDI-TOF MS at the proteomics core facilities at UiO (Thiede lab). An ULTRAFLEX II (Bruker Daltonics, Bremen, Germany) MALDI-TOF/TOF mass spectrometer was used after external calibration. The samples were mixed with matrix (20 mg/mL α-cyano-4-hydroxycinnamic acid in 0.3% aqueous trifluoroacetic acid/acetonitrile (1:1)) and applied to a stainless-steel sample holder. Basic settings of the MALDI-TOF/TOF instrument were as follows: Ion source 1, 25 kV; ion source 2: 21.85 kV; lens: 9.60 kV; reflector: 26.3 kV; reflector 2, 13.85 kV; deflector mode, polarity positive. MS spectra were transformed into peak lists by using the software FlexAnalysis version 2.4 (Bruker Daltonics, Bremen, Germany).

### Small-Angle X-ray Scattering

SAXS data were acquired on a Bruker NanoStar instrument using 40.0 μM H-GbpA-FL or 32.0 μM D-GbpA-FL in 100 mM NaCl, 20 mM Tris–HCl pH 8.0, with data acquisition times of 1 h per data set. Scattering intensities were recorded as a function of the scattering vector *q* = (4π/λ)sinθ, where 2θ is the scattering angle and λ is the wavelength (λ = 1.54 Å). Data were collected in the *q*-range: 0.009 to 0.3 Å^–1^.

The scattering intensities were corrected for electronic noise, empty cell scattering, and detector sensitivity. The scattering contribution from the buffer was subtracted, and intensities were calibrated to absolute units with H_2_O scattering as standard, using the *SUPERSAXS* program package (CLP Oliveira and JS Pedersen, unpublished; implementation explained in ref (25)).

Radii of gyration and pair-distance distribution functions (from inverse Fourier transform^26^) were calculated with *PRIMUS*(27) from the *ATSAS*(28) package. For both H-GbpA-FL and D-GbpA-FL, 20 low-resolution models were calculated by *ab initio* shape determination using the *DAMMIF*(29) software. We built average models with *DAMAVER*(30) and refined them with *DAMMIN.*(31) All three programs are from the *ATSAS*(28) package. SAXS data are summarized in Table 2.

**Table 2:** SAXS and SANS

(a) Sample details				
Organism		*Vibrio cholerae*		
Expression host		*E. coli* BL21(DE3) Star		
Uniprot sequence ID (residues in construct)		Q9KLD5 (24–485)		
Extinction coefficient [*A*_280_, 0.1% (=1 g/L)]		1.906		
*v̅* from chemical composition (cm^3^ g^–1^)		0.73		
Particle contrast, Δρ̅ (10^10^ cm^–2^)		2.99		
*MM* from chemical constituents (kDa)		51.3		
Protein concentration (mg/mL)		46.4 μM for H-GbpA, 36.0 for μM D-GbpA		
Solvent		100 mM NaCl, 20 mM Tris–HCl, pH 8.0		
				
(b) SAXS data-collection parameters				
Instrument		Bruker Nanostar with InCoatec Cu microsource and Våntec-2000 detector (RECX, University of Oslo, Norway)		
Wavelength (Å)		1.54		
Beam size (μm)		750 × 750		
Sample to detector distance (cm)		109		
*q* measurement range (Å^–1^)		0.00925–0.29866		
Absolute scaling method		Milli-Q water standard measurement		
Normalization		Transmitted intensities through semi-transparent beam-stop		
Exposure time (h)		1		
Capillary size (mm)		1.5		
Sample temperature		24 °C		
				
(c) Software employed for SAXS data processing, analysis and interpretation				
SAXS data processing		*SUPERSAXS* (CLP Oliveira and JS Pedersen, unpublished)		
Extinction coefficient estimate		ProtParam^32^		
Calculation of contrast and specific volume		*MULCh1.1*(33)		
Basic analysis		*PRIMUS* (*ATSAS*)^27,28^		
Shape reconstruction		*DAMMIF*(29)/*DAMAVER*^30^/*DAMMIN*^31^		
Representation		PyMOL		
				
(d) Structural parameters				
	H-GbpA		D-GbpA	
Guinier analysis				
*I*(0) (cm^–1^)	0.0406 ± 0.0008		0.03776 ± 0.0008	
*R*_g_ (Å)	37.35 ± 1.02		36.78 ± 1.25	
*q*_min_ (Å^–1^)	0.0114		0.0107	
*qR*_g_ max	1.26		1.24	
*R*^2^	0.92		0.88	
*MM* from *I*(0) (kDa) (ratio to predicted)	52.0 (1.01)		48.2 (0.94)	
*P*(*r*) analysis				
*I*(0) (cm^–1^)	0.0416 ± 0.0001		0.03944 ± 0.0007	
*R*_g_ (Å)	40.8 ± 1.0		40.6 ± 0.9	
*d*_max_ (Å)	142.5		142.5	
*q* range (Å^–1^)	0.0114–0.2109		0.0107–0.2165	
χ^2^	0.99		0.94	
Total quality estimate from *PRIMUS*	0.65		0.70	
*MM* from *I*(0) (kDa) (ratio to predicted)	53.2 (1.04)		50.5 (0.98)	
				
(e) Shape model-fitting results				
	H-GbpA		D-GbpA	
*DAMMIF*				
*q* range (Å^–1^)	0.0114–0.2109		0.0107–0.21086	
Symmetry, anisotropy assumptions	*P*1, None		*P*1, None	
NSD (Standard deviation)	0.695 (0.043)		0.811 (0.054)	
Resolution (Å)	31 ± 3		32 ± 3	
*MM* from *DAMMIF* (kDa) (ratio to predicted)	38.2 (0.74)		40.4 (0.79)	
χ^2^	0.984–1.003		0.797–0.804	
*DAMAVER*/*DAMMIN*				
*q* range	0.0114–0.2109		0.0107–0.21086	
Symmetry, anisotropy assumptions	*P*1, none		*P*1, none	
χ^2^	0.993		0.796	
Constant adjustment	Skipped		Skipped	
				
(f) SANS data analysis				
	H-GbpA 100% D_2_O	D-GbpA 0% D_2_O	D-GbpA 45% D_2_O	D-GbpA 100% D_2_O
Guinier analysis				
*I*(0) (cm^–1^)	0.075 ± 0.001	0.302 ± 0.004	0.118 ± 0.002	0.019 ± 0.001
*R*_g_ (Å)	36.2 ± 0.5	36.1 ± 0.7	34.7 ± 0.8	37.2 ± 2.5
*q*_min_ (Å^–1^)	0.0133	0.0159	0.0149	0.0181
*qR*_g_ max	1.26	1.22	1.30	1.18
*R*^2^	97.5	95.6	91.9	76.6

### Small-Angle Neutron Scattering

H-GbpA was dialyzed into a buffer containing 100 mM NaCl and 20 mM Tris–HCl pH 8.0 in 100% D_2_O. D-GbpA was dialyzed into the same buffer, but using different D_2_O/H_2_O ratios, i.e., at 100% H_2_O, 45% D_2_O, or 100% D_2_O. SANS data for the four samples were acquired at ILL beamline D11 at λ = 5.6 Å for 2 h, using a protein concentration of 39.2 μM. Subsequently, the data were processed with beamline software (Table 2).

### X-ray Crystallography

Hydrogenated and deuterated GbpA-D1 were crystallized using the same protocol and conditions. First, the proteins were saturated with Cu^2+^ by addition of CuCl_2_ in a molar ratio of 3:1 (CuCl_2_ to GbpA) and subsequently desalted to a buffer containing 100 mM NaCl, 20 mM Tris–HCl pH 8.0 using a 5 mL HiTrap desalting column. No crystals were obtained when the copper-binding step was omitted. Initial screening yielded crystals under many conditions, but most were either very small, irreproducible, or exclusive to either H-GbpA or D-GbpA. The following paragraph describes the procedure and conditions that yielded reproducible crystals in space group *P*2_1_2_1_2 for both proteins, which also diffracted to high resolution.

GbpA-D1 crystals grew from a solution containing the purified protein at 6–10 mg/mL. Sitting-drop vapor diffusion experiments were set up in 96-well 3-drop PS plates (SwissCI). 0.5 μL protein were added to an equal volume of crystallization solution containing 100 mM sodium cacodylate pH 6.5, 200 mM zinc acetate and 18% w/v PEG 8000 (VWR). Crystals grew over the course of two weeks at 20 °C. Crystals were subsequently cryoprotected in mother liquor supplemented with 15% glycerol and flash-cooled in liquid nitrogen before data collection.

Diffraction data were collected at the European Synchrotron Radiation Facility (ESRF, Grenoble, France) at the beamlines ID23–1 (Pilatus 6 M Dectris detector) for D-GbpA (diffraction to 1.1 Å) and ID23-2 (Pilatus3 X 2 M detector) for H-GbpA (diffraction to 1.6 Å resolution). The DOI for the data collection of H-GbpA-D1 is 10.15151/ESRF-ES-541149090 (no DOI was generated for the data collection of D-GbpA-D1).

X-ray data were processed automatically by the EDNA processing pipeline^34^ for H-GbpA-D1 and XIA2_DIALS^35^ for D-GbpA-D1. In all subsequent steps, the *CCP4* software suite^36^ was used. Structures were solved by molecular replacement (with *Phaser*(37)) using domain D1 of the published GbpA structure (PDB ID: 2XWX)^8^ as a model. Real-space refinement and model building were performed with *Coot*,^38^ and subsequent refinement cycles using *REFMAC5.*(39) Ions and water molecules were added only after the protein chain had been modeled. Finally, occupancies were refined for protein atoms and anomalous scattering ions. Zinc and copper ions were identified with confidence based on data collected at their absorption edges for anomalous scattering (Tables S1 and S2; Figure S3). Full anisotropic refinement was carried out for the 1.1 Å D-GbpA-D1 model, whereas this was not warranted for the lower-resolution H-GbpA-D1 structure (1.6 Å). The refined structures were deposited in the Protein Data Bank (PDB, www.rcsb.org)^40^ with PDB IDs: 8CC3 and 8CC5. Data collection and refinement statistics are reported in Table 3.

**Table 3:** Data Collection and Refinement Statistics^a^

	H-GbpA D1	D-GbpA D1
(a) Data collection		
Beamline	ID23–2 (ESRF)	ID23–1 (ESRF)
Wavelength (Å)	0.8731	0.9763
Resolution range	44.7–1.6 (1.68–1.62)	47.1–1.1 (1.17–1.13)
Space group	*P*2_1_2_1_2	*P*2_1_2_1_2
Unit cell axes: a, b, c (Å)	75.3 89.4 47.5	74.9 89.1 47.1
*R*_merge_ (%)	11.4 (>100)	7.9 (>100)
CC_1/2_	1.00 (0.37)	1.00 (0.22)
Mean *I*/σ	10.8 (0.9)	6.1 (1.2)
Completeness (%)	99.9 (100.0)	98.7 (95.9)
Multiplicity	6.8 (6.7)	2.2 (2.0)
Unique reflections^b^	78,744 (4085)	221,328 (11391)
(b) Refinement		
Resolution range (Å)	44.7–1.6	47.1–1.1
*R*_work_/*R*_free_^c^	0.182/0.217	0.184/0.201
Macromolecules/a.u.	2	2
Number of non-hydrogen atoms	3240	3526
Protein	3034	3256
Ligands	19	18
Waters	187	252
*B*-factors (Å^2^)		
Protein	27.0	12.6
Ligands	36.0	14.6
Waters	32.1	22.0
R.m.s.d. from ideal values		
Bond length (Å)	0.008	0.009
Bond angles (°)	1.44	1.58
Ramachandran Plot		
Favored (%)	95.5	96.7
Outliers (%)	0.3	0.0
PDB ID	8CC3	8CC5

a Statistics for the highest resolution shell shown in parentheses.

b Data reported treating Bijvoet pairs as separate reflections.

c *R*_free_ was calculated from 5% of randomly selected reflections for each dataset.

To unambiguously identify the metal species observed in GbpA-D1, diffraction data were collected at the K absorption edge of zinc (around 9660 eV) and copper (around 8980 eV). Three data sets were collected at the BioMAX beamline of MAX IV (Lund, Sweden; Table S1) using isomorphous crystals grown under the same conditions as the H-GbpA-D1 and D-GbpA-D1 structures described above. The data were integrated and scaled by the *autoPROC* automatic processing pipeline at MAX IV,^41^ and subsequently scaled and truncated to 2.5 Å with *XSCALE*, a component of the *XDS* software package.^42^ The phases for the highest-resolution dataset (9320 eV) were determined by molecular replacement as described above, and the structure thereafter refined in iterative cycles of maximum-likelihood refinement using *REFMAC5*(39) and manual real-space refinement in *Coot*.^38^ Data collection and refinement statistics for this dataset are given in Table S2.

Phase information from the 9320 eV refined structure was used to generate anomalous difference maps for each of the datasets (*D*_ano_^10.0k^, *D*_ano_^9.3k^, *D*_ano_^8.5k^) using the *FFT* tool from the *CCP4* program suite.^36^ Completeness of the datasets at a resolution higher than 3 Å was limited due to the presence of water ice (see Table S1); however, the anomalous signal at lower resolution was sufficient to allow identification of the positions and identities of the metal ions. Difference density maps of anomalous difference maps were generated using the *CAD* and *FFT* tools to find peaks corresponding to copper atoms (*D*_ano_^9.3k^ – *D*_ano_^8.5k^) and zinc atoms (*D*_ano_^10.0k^ – *D*_ano_^9.3k^). For each metal-binding site detected by any of the anomalous difference maps (*D*_ano_^10.0k^, *D*_ano_^9.3k^, *D*_ano_^8.5k^), the combined presence or absence of a peak in the *D*_ano_^9.3k^ – *D*_ano_^8.5k^ and the *D*_ano_^10.0k^ – *D*_ano_^9.3k^ maps revealed whether the position was occupied by copper, zinc, or a combination of both. The histidine brace motif only showed a peak in the *D*_ano_^9.3k^ and the *D*_ano_^9.3k^ – *D*_ano_^8.5k^ maps (copper absorption edge) but not in the *D*_ano_^10.0k^ – *D*_ano_^9.3k^ map calculated around the zinc absorption edge. This confirmed the exclusive presence of copper in its catalytic position.

### Activity Studies

GbpA was saturated with Cu^2+^ as described in the previous section, and β-chitin nanofibers from France Chitine (Orange, France) were prepared according to a protocol developed by Loose et al.^9^ 1 μM GbpA-FL was mixed with 5 mg/mL β-chitin nanofibers in 20 mM Tris–HCl pH 8.0, and the reaction initiated by addition of 1 mM sodium ascorbate. After incubation on an INFORS shaker for 1.5 h at 37 °C, the reaction was stopped by boiling and subsequent filtering through a 0.2 μm cellulose filter. The products were measured with MALDI-TOF MS. The experiment was performed in triplicates for both H-GbpA and D-GbpA.

## Results and Discussion

### High-Yield Production of Deuterated GbpA

In order to optimize expression conditions for GbpA, we first carried out experiments using non-deuterated modified M9 medium, which we refer to as M9glyc+ (Table 1; recipe modified from refs (19,43)). A plateau in optical density at 600 nm (OD_600_) was reached at 12 AU after 12 h without induction (Figure S1A). For comparison, expression in LB medium reached a plateau much earlier (after 5 h) but only at 4 AU (Figure S1A). Expression of GbpA-FL in M9glyc+ was induced by adding 1 mM IPTG at four different cell densities, OD_600_ = 0.7, 3.0, 7.2, and 11.0 (Figure S1B). The optimal induction point was identified to be at OD_600_ = 3.0 based on band intensities over background on SDS-PAGE (Figure 1A). The protein was subsequently purified using a periplasmic isolation protocol, followed by AEX and SEC. The protein eluted at the same retention volume as GbpA-FL expressed in TB (Figure S1C) and exhibited equivalent purity (Figure S1D).

We then grew cell cultures containing GbpA-FL-encoding plasmid in deuterated M9glyc+ medium (Table 1), following a stepwise adaptation protocol, from LB/H_2_O to LB/D_2_O to D_2_O and glycerol-d_8_-containing minimal medium (Figure 2). In the final step, cells were grown to OD_600_ = 3.0, and expression was induced. Both H-GbpA-FL and D-GbpA-FL eluted as a single peak from SEC and were shown to be highly pure as evaluated by SDS-PAGE (Figure 1B–D).

We found that using deuterated glycerol instead of the more expensive deuterated glucose used by Cai et al.^19^ worked well. A protein yield of 12 mg purified protein from 1 L of expression media was calculated from absorbance at 280 nm. Surprisingly, the yield was approximately two-fold higher than from optimized expression in non-deuterated TB medium, as suggested by final protein contents of the purified samples as compared to the total volume of the expression cultures.

Subsequently, we applied the same protocol to GbpA-D1, in preparation of NMX experiments. A protein yield of 3 mg pure deuterated protein from 1 L of expression media was obtained, as calculated from absorbance at 280 nm (thus 4-fold less than for D-GbpA-FL). Purity was assessed with SDS-PAGE (Figure 1D). The protocol, with small variations, was subsequently scaled up with improved yields by D-lab at ILL and DEMAX at ESS. The procedure for producing deuterated proteins used at DEMAX (ESS) is done in traditional shaker flasks and includes supplementing the deuterated glycerol in the media with deuterated algal extracts. The method described in this work serves as a proof-of-concept, and the optimized formulation and procedure is the subject of a separate publication that is in preparation. The procedures at D-Lab (ILL) use fermenters and as such require far less D_2_O (almost 3-fold less) than the shaker method, but the batch-fed approach uses more deuterated glycerol. Despite the fundamental differences in how the deuterium labeling is achieved, both D-Lab and DEMAX produced around 30 mg of D-GbpA-D1 each, indicating that the methods are equivalent for this protein.

An additional difference that could potentially affect the protein quality and yield was the handling after the protein production stage. Usually, the cells were immediately further processed for periplasmic protein extraction, but in one case, the cell paste was frozen and shipped prior to further extraction. This step may have reduced the final yield, but the purified protein could be crystallized, as reported below, suggesting that the sample quality did not suffer significantly.

### D-GbpA Is Fully Deuterated, Catalytically Active and Retains Its Fold Compared to H-GbpA

**Figure 3:**
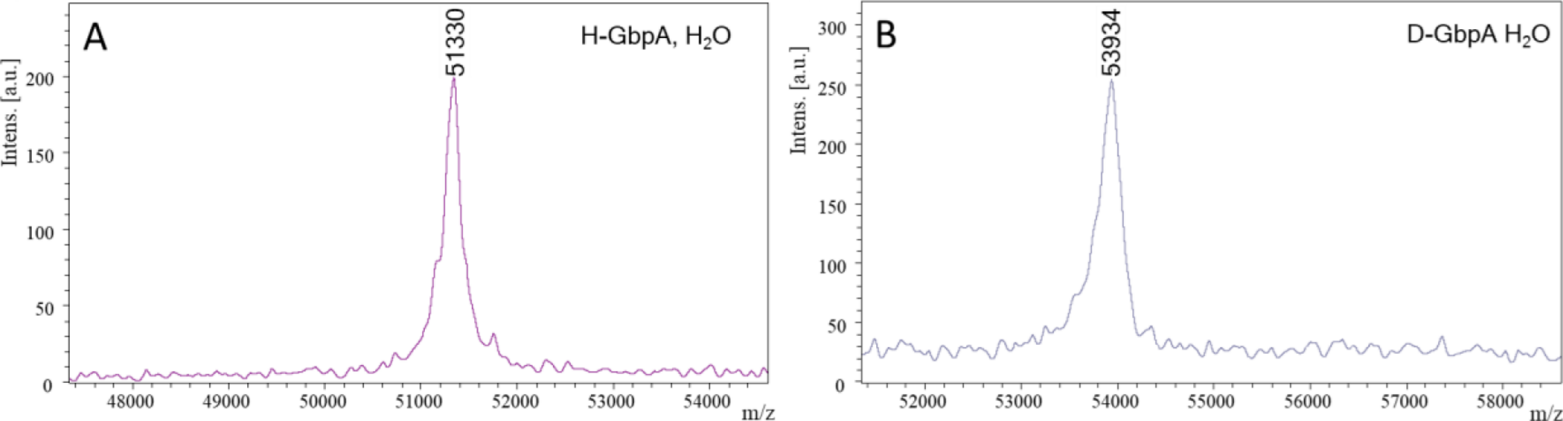
Deuteration. The deuteration levels were quantified with MALDI-TOF MS for full-length H-GbpA (A) and D-GbpA (B), in H_2_O. With a theoretical hydrogenated mass of 51,248 Da and a theoretical per-deuterated mass of 53,930 Da, the deuteration level of D-GbpA was determined to be approximately 97%. The slightly higher experimental values compared to theoretical values are likely due to instrument calibration for different mass ranges.

To assess the level of deuteration, intact mass of the full-length protein was quantified by MALDI-TOF Mass Spectrometry (MS) (Figure 3). Masses of non-deuterated (H-GbpA) and deuterated GbpA (D-GbpA) in H_2_O were measured to be 51,330 and 53,934 Da, respectively. The theoretical molecular weight of a deuterated protein (MW_dT_) in H_2_O is given by
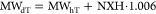
where MW_hT_ is the theoretical molecular weight of the non-deuterated protein, NXH the number of non-exchangeable hydrogens, and 1.006 is the relative mass difference between hydrogen and deuterium. Using the method of Meilleur et al.^44^ for calculating the number of non-exchangeable hydrogens and estimating deuteration levels gives relative molecular weight values of MW_hT_ = 51,250 and MW_dT_ = 53,930 for GbpA. The deuteration level can then be calculated with
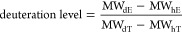
where MW_dE_ and MW_hE_ correspond to the experimentally determined masses of the deuterated and non-deuterated GbpA, respectively. This gives a deuteration level of 97%.

The remaining 3% undesired hydrogen may result from vapor exchange with the air or contamination from the various chemicals used (Table 1). However, 97% deuteration is more than sufficient for neutron-based experiments, and the neutron SLD of 6.12 × 10^–6^ Å^–2^ (in H_2_O) for D-GbpA (compared to 1.97 × 10^–6^ Å^–2^ for H-GbpA) is expected to be significantly distinguishable from all non-deuterated biomolecules. Interaction studies exploiting contrast matching should therefore be possible with any interaction partner with a defined SLD, most obviously chitin or mucin, but also potential bacterial cell-surface interaction partners or enzyme complex partners yet to be identified.

**Figure 4:**
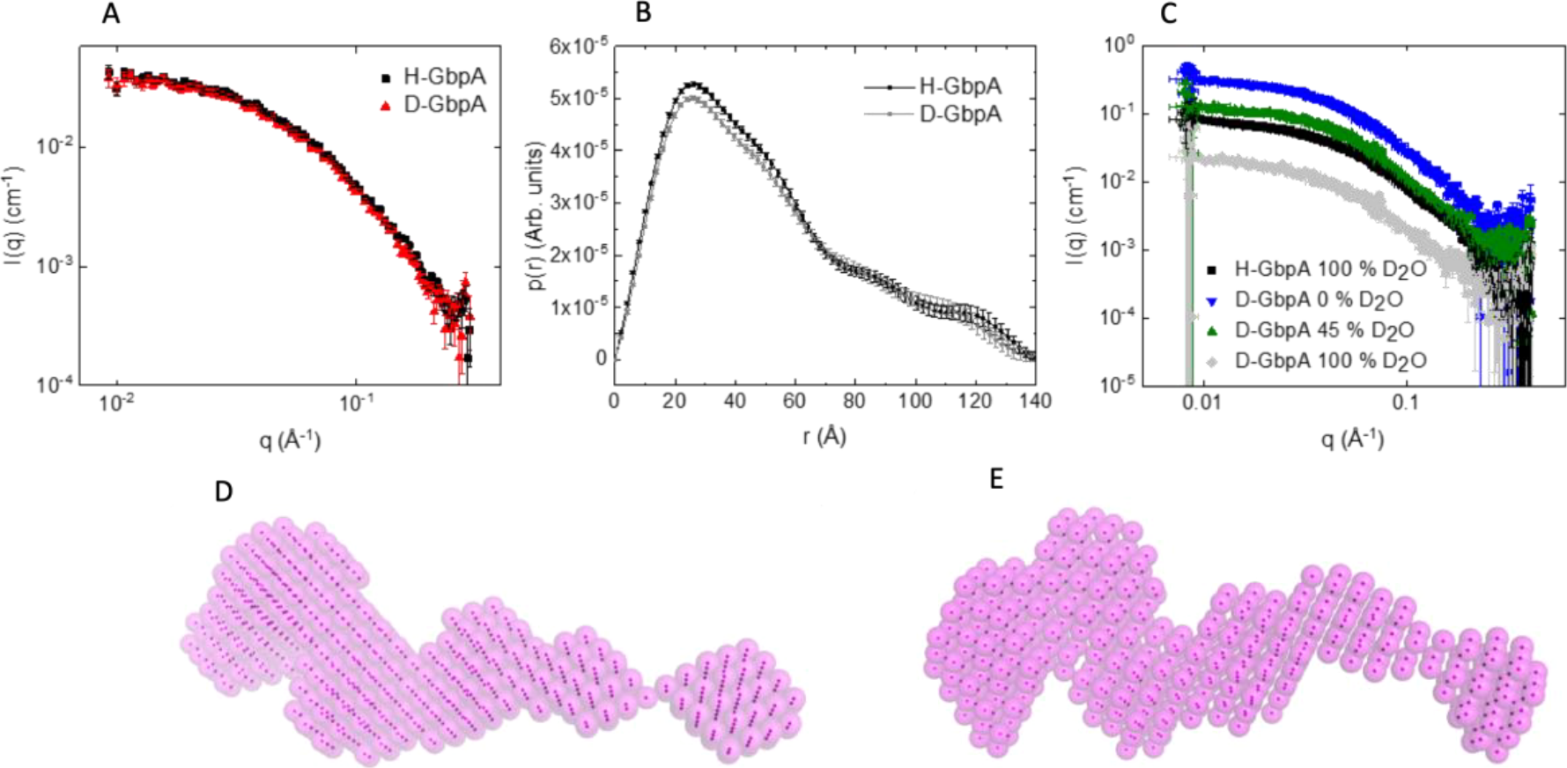
Small-angle scattering experiments on H-GbpA and D-GbpA. (A) SAXS curves of full-length H-GbpA and D-GbpA (intensities normalized to a 1 mg/mL concentration. (B) Pair-distance distribution functions, revealing elongated proteins with maximal dimension of 140 Å. (C) SANS data for H-GbpA and D-GbpA, in buffers containing different levels of D_2_O. (D, E) SAXS *ab initio* models averaged from 20 structural models, shown for H-GbpA (D) and D-GbpA (E), respectively.

To confirm that the overall structure of GbpA-FL was unaffected by deuteration, we performed SAXS experiments for both D-GbpA and H-GbpA (Figure 4A). Indeed, the SAXS profiles of both proteins were highly similar, with a radius of gyration (*R*_g_) of 37.4 Å for H-GbpA and 36.8 Å for D-GbpA. Both proteins are monomers in solution, with a maximal diameter of approximately 140 Å (Figure 4B). Structural parameters are summarized in Table 2. *Ab initio* modeling suggests similar, elongated structures of both hydrogenated and deuterated GbpA (Figure 4D,E). In order to test the neutron scattering of D-GbpA at different concentrations of D_2_O, we carried out SANS experiments on D-GbpA under three different conditions (Figure 4C), comparing the scattering in buffer at 100% H_2_O with 45% D_2_O (around the match point of non-deuterated proteins and carbohydrates) and 100% D_2_O (where perdeuterated proteins should have the lowest scattering, since the match point is above 100%). We saw that the form factor of D-GbpA was fairly consistent over the three different D_2_O concentrations and similar to that of H-GbpA, with comparable *R*_g_ for all conditions. We also confirmed that D-GbpA scattered well at 45% D_2_O and that the D-GbpA match point was above 100% D_2_O, i.e., that D-GbpA could not be matched out even at 100% D_2_O due to its high deuteration level. Parameters from SANS are summarized in Table 2.

**Figure 5:**
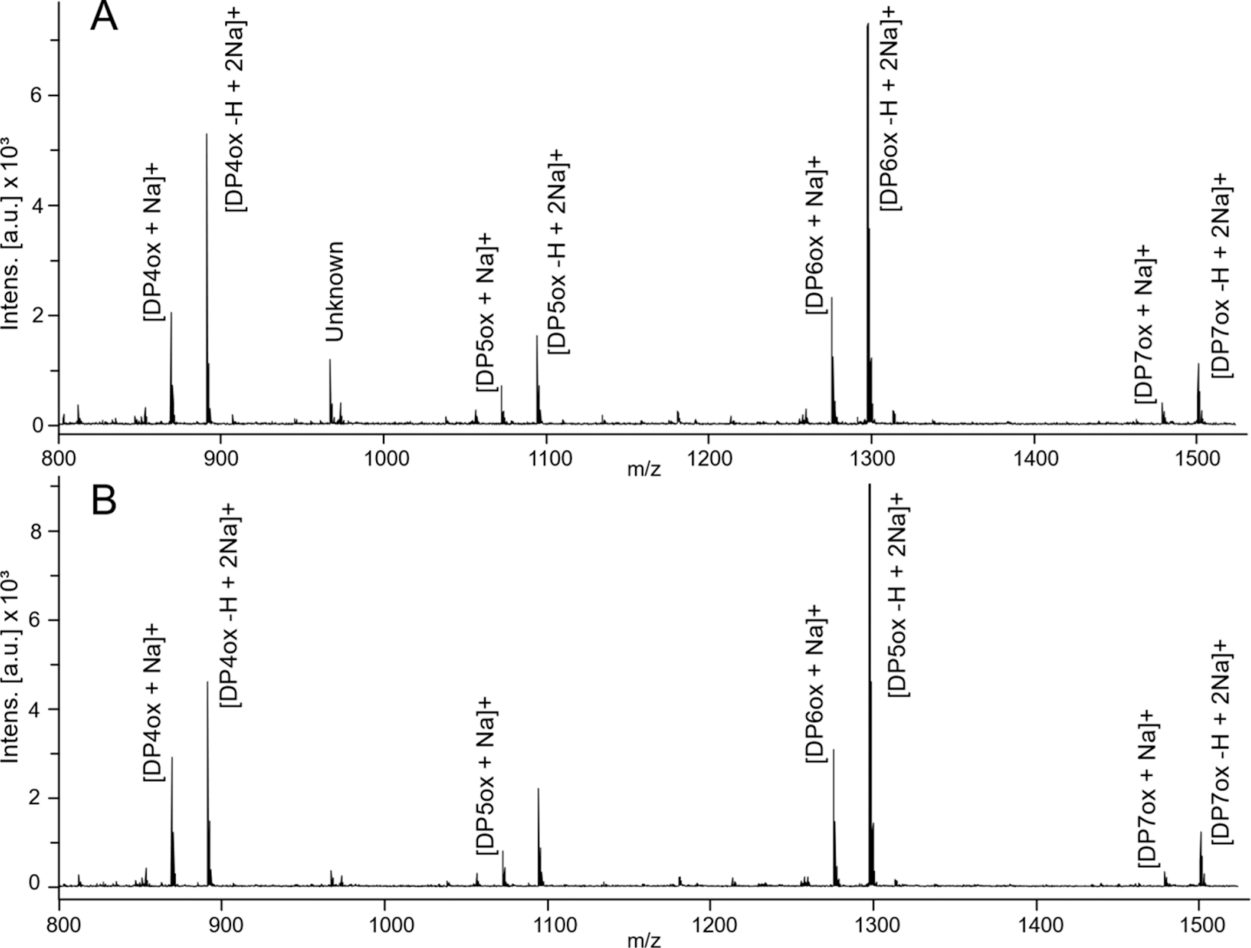
LPMO activity. MALDI-TOF MS spectra of LPMO reaction products after cleavage of β-chitin by H-GbpA (A) and D-GbpA (B). Labeled peaks correspond to chitooligosaccharides of four (869–891), five (1073–1095), six (1276–1298), and seven (1501) units. All labeled peaks correspond to masses of oxidized chitooligosaccharides. Some peaks correspond to saccharides bound to one sodium ion (1276, 1073, 869) and others to two (671, 891, 1095, 1298, 1501). One significant peak is an unknown species (967).

In order to verify that the catalytic activity was unaffected by deuteration, LPMO activity towards β-chitin was assessed by measuring the masses of chitooligosaccharide products by MS (Figure 5). H-GbpA and D-GbpA yielded the same products, thus activity was maintained after deuteration. We refer to Loose et al.^9^ for a more in-depth study of the activity of GbpA and the components involved in catalysis.

### Crystal Structures of GbpA-D1 Show no Differences upon Deuteration

The LPMO domains (D1) of both deuterated and non-deuterated GbpA were crystallized (Figure S2), and X-ray diffraction data were collected. Both proteins crystallized in space group *P*2_1_2_1_2 with very similar cell parameters. *P*2_1_2_1_2 is a relatively high-symmetry space group, which is preferred for NMX, as less angular data are needed to obtain complete data sets. X-ray crystal structures were determined to 1.6 Å resolution and 1.1 Å resolution for H-GbpA-D1 and D-GbpA-D1 (sample produced at ILL), respectively. The high resolution for D-GbpA is especially promising, as crystal packing and diffraction quality are important for NMX.

**Figure 6:**
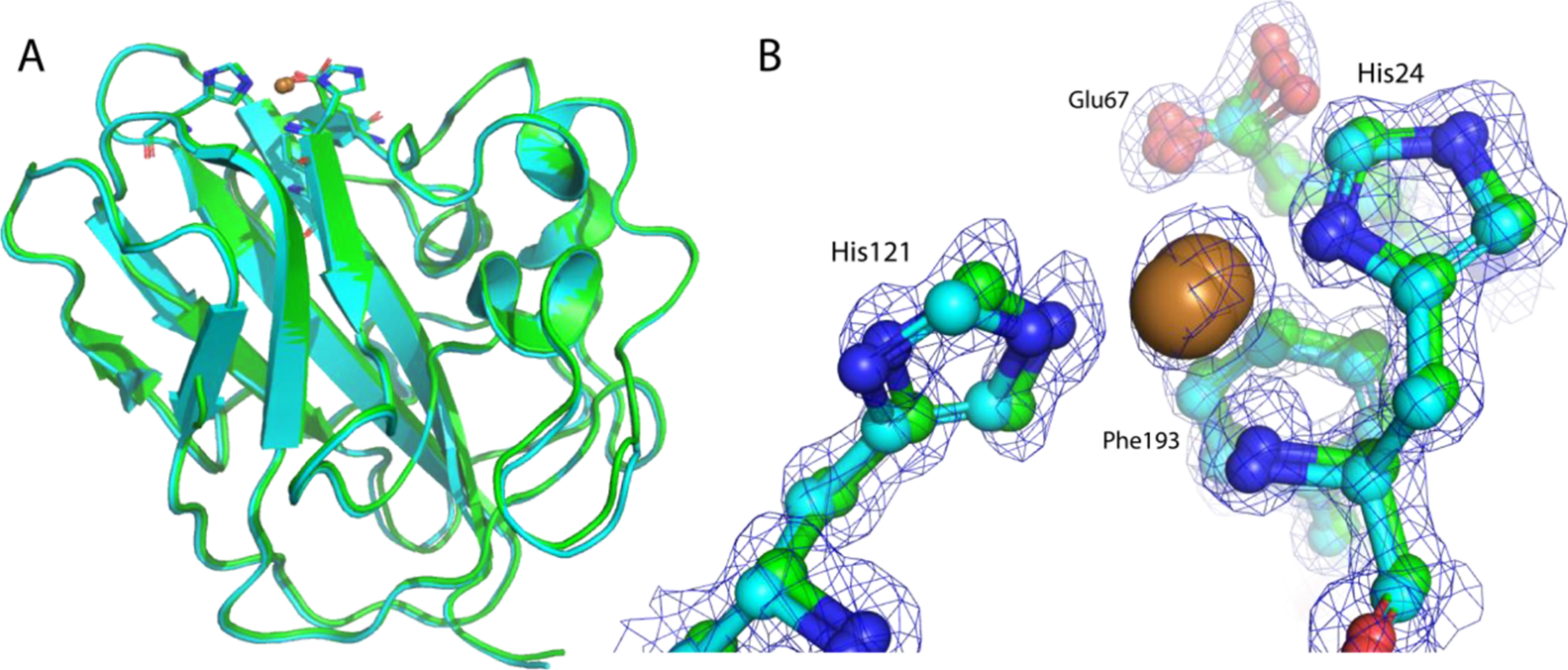
H-GbpA-D1 and D-GbpA-D1 structures. (A) Superimposition of X-ray crystal structures of D-GbpA-D1 (green; PDB ID: 8CC5) and H-GbpA-D1 (cyan, PDB ID: 8CC3) (r.m.s.d. between C_α_ atoms = 0.2 Å, as calculated with PyMOL, Schrödinger LLC). Both structures are the results from this work. (B) Close-up view of active sites shown in A, including histidine brace and copper ions (bronze spheres), with sigma-A-weighted 2*mF*_o_ – *DF*_c_ electron density map shown for D-GbpA-D1 at 1.5 σ. Figures were prepared with PyMOL (Schrödinger LLC).

Both proteins were crystallized in the catalytically active, copper-bound states (Figure 6B), differing from the crystal structure of GbpA (domains 1–3; PDB ID: 2XWX) from Wong et al.,^8^ which lacked the copper ion in the active site. Analysis of anomalous scattering from data sets collected at the absorption edges of copper and zinc confirmed the active site to be exclusively occupied by copper (Figure S3 and Tables S1 and S2). The conformation of the histidine brace, which is very similar among copper-free and copper-bound LPMO structures in the Protein Data Bank (see, e.g., PDB IDs: 6IF7,^45^6RW7,^46^ and 5FTZ(47)), is conserved in our structures. Table 3 summarizes the data collection and refinement statistics. Superimposition of the deuterated and hydrogenated GbpA-D1 structures yielded r.m.s.d. values of appr. 0.2 Å for C_α_ atoms (Figure 6), showing that the fold is not affected by deuteration. In addition, when comparing our X-ray structures with the one from Wong et al.^8^ for domains 1–3 of GbpA, the r.m.s.d. values are below 0.3 Å. The crystallization condition thus serves as a good starting point for further optimization for NMX.

## Conclusions

Neutron scattering techniques have great potential to offer deep insights into the molecular structure and function of proteins in complex environments, providing an important complement to other structural biology techniques. Here, we have demonstrated the feasibility of perdeuteration for GbpA, a bacterial colonization factor with LPMO activity, resulting in yields and deuteration levels highly compatible with NMX and SANS without the need for specialized fermentation equipment. We report a new deuteration protocol based on algal extracts and showed that the protein investigated remains structurally uncompromised and active. In addition to first SANS studies of full-length GbpA (GbpA-FL), we succeeded in obtaining well-diffracting crystals of the GbpA LPMO domain (GbpA-D1) in both the deuterated and non-deuterated states. Whereas production of 70% deuterated LPMOs has been achieved before and used for SANS interaction studies,^48^ this is to our knowledge the first time that perdeuteration of an LPMO has been achieved, and a more in-depth study of the effect of deuteration of LPMOs has been performed.

This work, alongside numerous other neutron crystallographic studies, further demonstrates the feasibility of NMX and the important role that perdeuteration plays in optimizing the quality of results. Although large crystals are needed for NMX, and optimization of crystal growth is still required, the possibility of using deuterated protein can reduce the volume requirements by up to a factor of 10. With better NMX structures of LPMOs, it is anticipated that the protonation states of key amino acids in and around the active sites will be revealed and that the importance of the water network within the LPMOs can be understood. Given the recently solved crystal structure of full-length VhLPMO10A from*V. campbellii*, a close homologue of GbpA, NMX studies of FL-GbpA and homologues may also become a possibility.^11^ Neutron studies with SANS or NR can reveal how LPMOs interact with the carbohydrate substrates and how the LPMO structure adapts to carbohydrate surfaces or fibers.

Our results will thus further enable neutron-based studies of perdeuterated GbpA and LPMOs in general. In addition, other techniques that benefit from isotope labeling, such as NMR spectroscopy, may also benefit from the labeling procedure presented here.

## Supporting Information Available

GbpA expression in non-deuterated M9glyc+ medium; LPMO crystals; metal ion identification by anomalous diffraction analysis; anomalous data collection parameters for the characterization of anomalous scatterers in H-GbpA-D1 crystals; and X-ray data collection and refinement statistics for H-GbpA-D1 at 9,320 eV (PDF)

## Accession Codes

UniProt ID: Q9KLD5 (Gene gbpA). PDB IDs: 8CC3 and 8CC5.

## Abbreviations

AEX: anion-exchange chromatography
EDTA: ethylenediaminetetraacetic acid
ESRF: European Synchrotron Radiation Facility
ESS: European spallation source
GbpA: *N*-acetylglucosamine-binding protein A
D-GbpA: deuterated GbpA
*d*_max_: maximum dimension
H-GbpA: hydrogenated (i.e., non-deuterated) GbpA
ILL: Institut Laue-Langevin
IPTG: isopropyl β-d-1-thiogalactopyranoside
LB: Luria Bertani
LPMO: lytic polysaccharide monooxygenase
MALDI-TOF: Matrix-Assisted Laser Desorption/Ionization-Time Of Flight
MM: molecular mass
MS: mass spectrometry
NMR: nuclear magnetic resonance
NMX: neutron macromolecular crystallography
NR: neutron reflectometry
NSD: normalized spatial discrepancy
OD: optical density
PMSF: phenylmethylsulfonyl fluoride
*R*_g_: radius of gyration
SANS: small-angle neutron scattering
SAXS: small-angle X-ray scattering
SDS-PAGE: sodium dodecyl sulphate–polyacrylamide gel electrophoresis
SEC: size-exclusion chromatography
SLD: scattering-length density
TB: terrific broth
Tris: tris(hydroxymethyl)aminomethane
