# Supplementary material for: Perdeuterated GbpA Enables Neutron Scattering Experiments of a Lytic Polysaccharide Monooxygenase
Source: ACS Omega. 2023 Jul 31;8(32):29101–12. doi: 10.1021/acsomega.3c02168 (PMC10433351; doi:10.1021/acsomega.3c02168)
Supplement: Supplementary file 1 — ao3c02168_si_001.pdf [file ao3c02168_si_001.pdf]

SUPPORTING INFORMATION

Perdeuterated GbpA enables neutron scattering experiments of a lytic polysaccharide monooxygenase

Henrik Vinther Sørensen[1§], Mateu Montserrat-Canals[1,2], Jennifer S. M. Loose[3], Zoe Fisher[4,5], Martine Moulin[6], Matthew P. Blakeley[7], Gabriele Cordara[1], Kaare Bjerregaard-Andersen[1,§], Ute Krengel[1*]

[1] Department of Chemistry, University of Oslo, NO-0315 Oslo, Norway

[2] Centre for Molecular Medicine Norway, University of Oslo, NO-0318 Oslo, Norway

[3] Faculty of Chemistry, Biotechnology and Food Science, Norwegian University of Life Sciences (NMBU), NO-1340 Ås, Norway

[4] Science Directorate, European Spallation Source ERIC, P.O. Box 176, SE-221 00 Lund, Sweden

[5] Department of Biology, Lund University, 35 Sölvegatan, SE-223 62 Lund, Sweden

[6] Life Sciences Group, Institut Laue-Langevin, 71 avenue des Martyrs, 38042 Cedex 9, Grenoble, France

[7] Large-Scale Structures group, Institut Laue-Langevin, 71 avenue des Martyrs, 38042 Grenoble, France

[§] Present addresses: Henrik V. Sørensen, Division of Computational Chemistry, Lund University, SE-223 62, Sweden; Kaare Bjerregaard-Andersen,Ottilia vej 9, H. Lundbeck A/S, DK-2500 Valby, Denmark

[*]Correspondence: Ute Krengel (ute.krengel@kjemi.uio.no; +47-22855461)

LIST OF MATERIAL INCLUDED:

Figures S1-S3 (S1, GbpA expression in non-deuterated M9glyc+ medium; S2, LPMO crystals; S3, Metal ion identification by anomalous diffraction analysis)

Tables S1-S2 (S1, Anomalous data collection parameters for the characterization of anomalous scatterers in H-GbpA-D1 crystals; S2,X-ray data collection and refinement statistics for H-GbpA-D1 at 9,320 eV)

SUPPORTING FIGURES

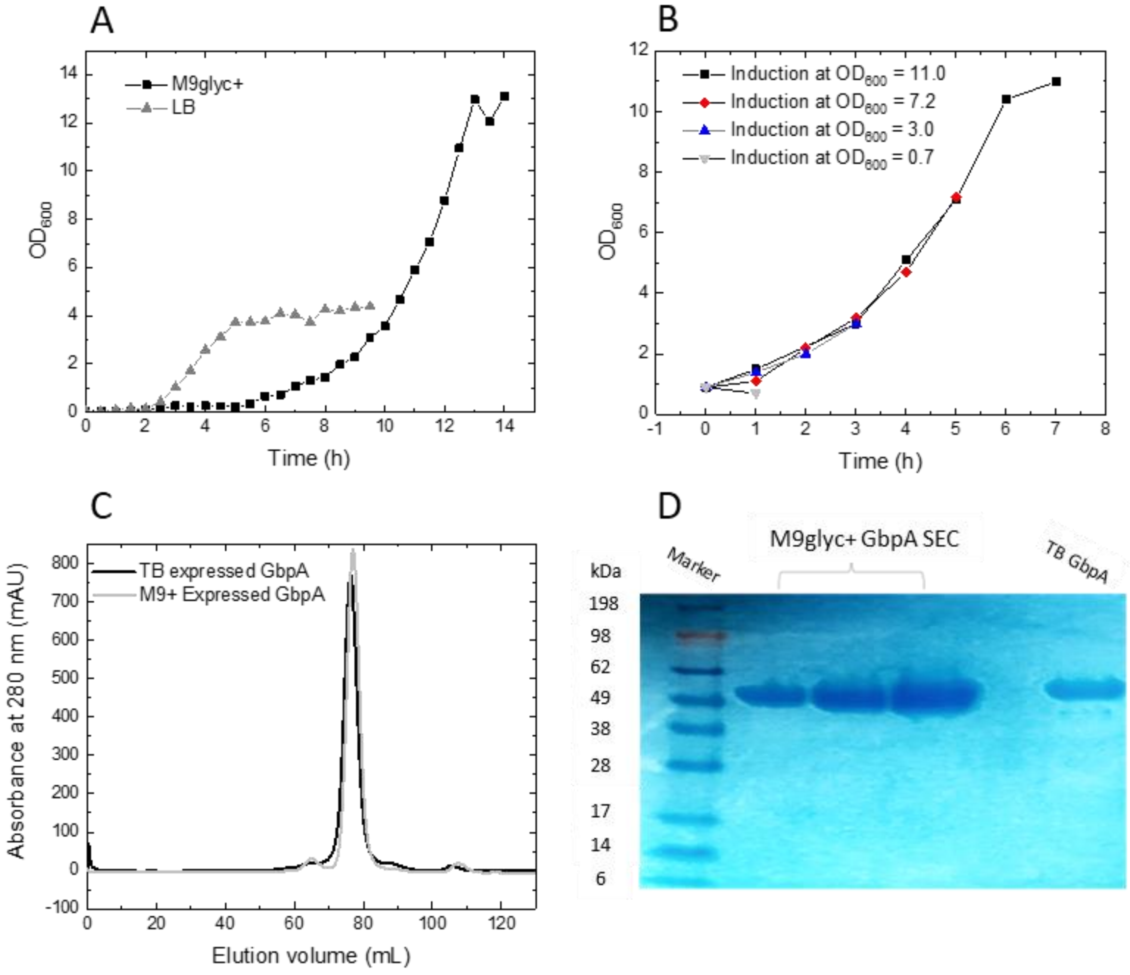

Figure S1. GbpA expression in non-deuterated M9glyc+ medium. A Growth curves for *E. coli* BL21(DE3) cells containing GbpA-FL-encoding plasmid. Luria Bertani (LB) compared to minimal medium (non-deuterated M9glyc+). **B** Growth curves in minimal medium (non-deuterated M9glyc+) up to different induction points. All cultures were “boosted” by LB pre-cultures. Expression was initiated by the addition of IPTG at four different optical densities. **C** SEC elution profile for GbpA expressed in TB or minimal media show comparable elution profiles and retention volumes. **D** SDS-PAGE of GbpA expressed in TB or minimal media, both showing high purity. Marker: SeeBlue plus 2.

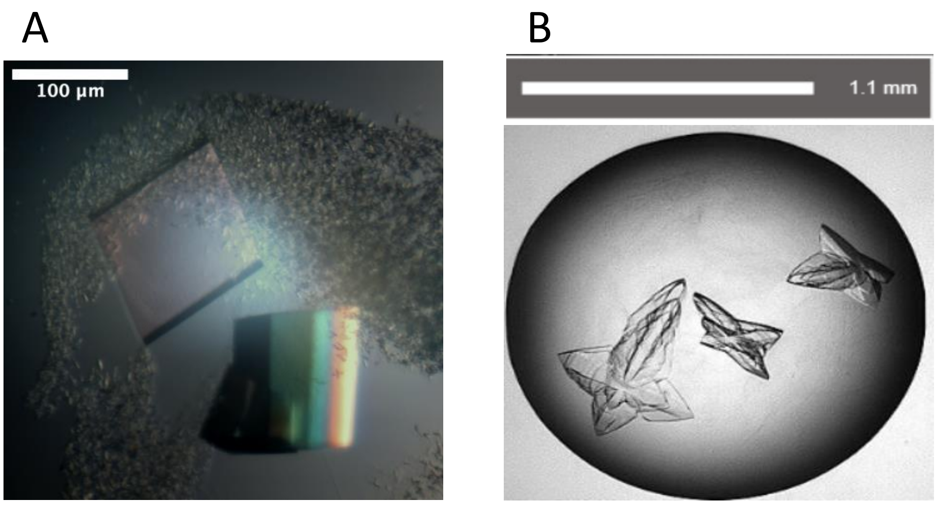

Figure S2: LPMO crystals. Examples of crystals of H-GbpA-D1 (**A**) and D-GbpA-D1 (**B**), corresponding to the same crystallization conditions and space group.

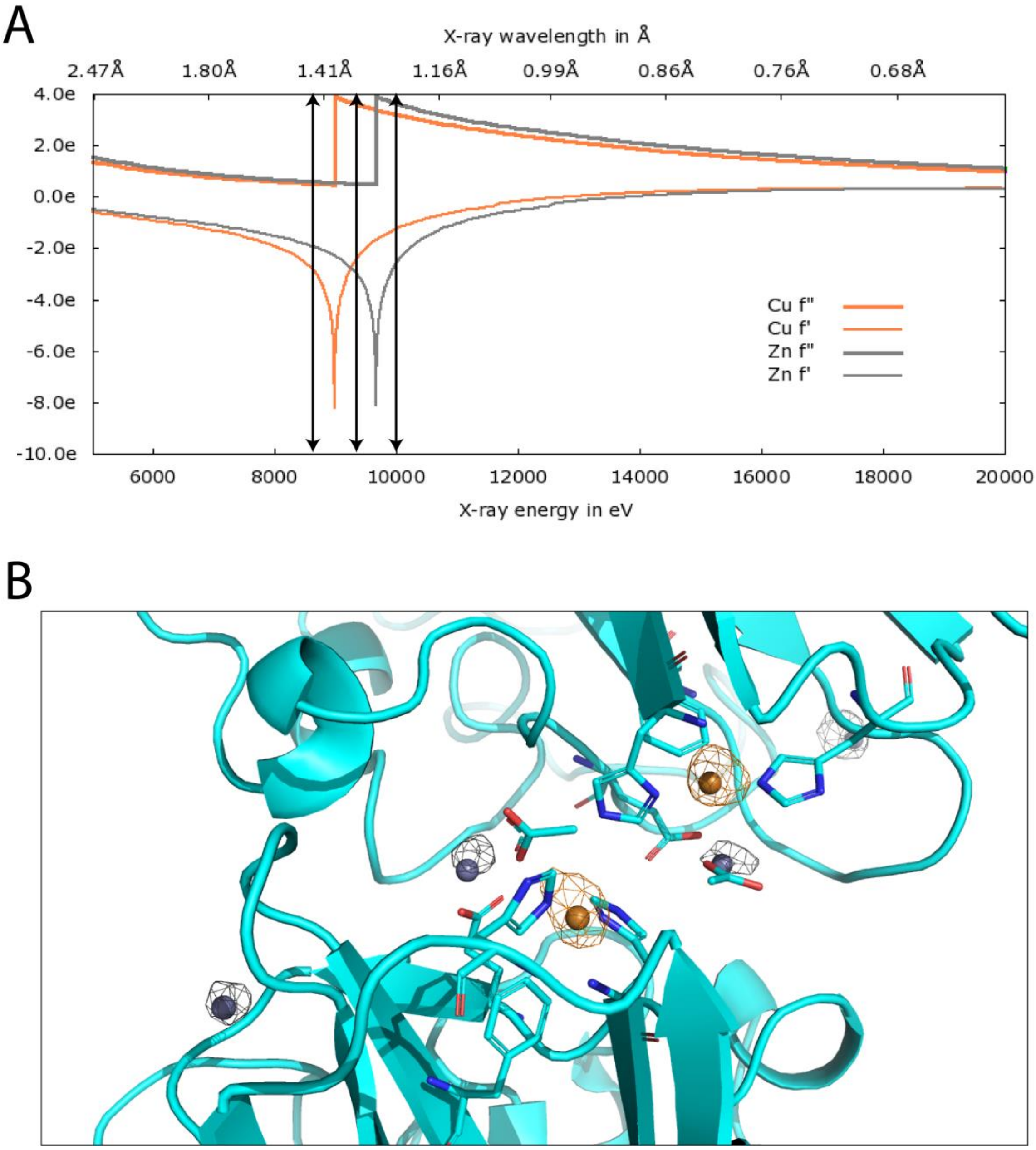

Figure S3: Metal ion identification by anomalous diffraction analysis. A X-ray anomalous scattering coefficients (f´and f´´) for copper and zinc. The energies around the absorption edges at which datasets were collected are marked with arrows. Generated with *http://skuld.bmsc.washington.edu/scatter/.* **B** Cartoon representation of the interface of the two H-GbpA-D1 molecules in the asymmetric unit. Catalytically relevant residues and acetate ions are shown in stick representation. Copper ions are shown in bronze, zinc ions are colored grey. Bronze mesh identifying copper corresponds to positive difference density when comparing difference Fourier maps for anomalous scattering around the absorption edges of copper ($D_{ano}^{9.3k}$-$D_{ano}^{8.5k}$), whereas the densities in grey correspond to positive difference density when comparing difference Fourier maps for the anomalous scattering around the absorption edges of zinc ($D_{ano}^{10.0k}$-$D_{ano}^{9.3k}$). A cut-off of 4σ was used for both maps. The analysis allowed the unambiguous identification of copper in the histidine brace motif of the LPMO active site.

Table S1. Anomalous data collection parameters for the characterization of the anomalous scatterers in H-GbpA-D1 crystals

(a) Data collection and processing			
	H-GbpA-D1$^{10.0k}$	H-GbpA-D1$^{9.3k}$	H-GbpA-D1$^{8.5k}$
Beamline	BioMAX (MAX IV)	BioMAX (MAX IV)	BioMAX (MAX IV)
Wavelength (Å)	1.2398	1.3303	1.4507
Resolution [a]	11.2-2.5 (2.56-2.50)	11.2-2.5 (2.56-2.50)	11.2-2.5 (2.56-2.50)
$CC_{1/2}$	0.99 (0.89)	0.99 (0.92)	0.99 (0.91)
Mean I/σ	13.4 (4.7)	10.9 (4.8)	13.0 (5.3)
Completeness (%)	92.7 (99.6)	88.6 (99.7)	98.8 (98.4)
Unique reflections [b]	18733 (1466)	17905 (1467)	19983 (1449)
Anomalous correlation	0.40 (0.23)	0.31 (0.10)	0.11 (0.11)
Anomalous signal	1.19 (0.83)	1.03 (0.72)	0.84 (0.75)
Anomalous unique reflections	8259 (656)	7816 (669)	8869 (653)

[a] Resolution range used for the generation of anomalous difference maps after scaling and truncation with *XSCALE*[38]
[b] Data reported treating Bijvoet pairs as separate reflections
Statistics for the highest resolution shell are shown in parentheses.

Table S2. Data collection and refinement parameters for H-GbpA-D1 at 9,320 eV

(a) Data collection

	H-GbpA-D1
Beamline	BioMAX (MAX IV)
Wavelength (Å)	1.2398
Resolution range (Å)	43.2 – 2.0 (2.08 – 2.01)
Space group	$P\ 2_1 2_1 2$
Unit cell axes: a, b, c (Å)	74.8 86.4 46.8
R_{merge} (%)	12.5 (48.9)
$CC_{1/2}$	0.99 (0.80)
Mean I/σ	7.7 (1.12)
Completeness (%)	79.4 (10.5)
Multiplicity	3.3 (2.1)
Unique reflections [a]	30689 (214)

Statistics for the highest resolution shell shown in parenthesis

(b) Refinement

Resolution range (Å)	43.2 – 2.0
R_{work}/ R_{free} [b]	0.257/0.325
Macromolecules/a.u.	2
Number of non-hydrogen atoms	2858
Protein	2837
Ligands	2
Waters	19
B-factors (Å^2)	
Protein	42.8
Ligands	49.0
Waters	34.9
R.m.s.d. from ideal values	
Bond length (Å)	0.014
Bond angles (deg)	1.96
Ramachandran Plot	
Favored (%)	93.0
Outliers (%)	0.6

[a] Data reported treating Bijvoet pairs as separate reflections

[b] R_{free} was calculated from 5% of randomly selected reflections for each dataset. The quality of the data was limited due to the presence of ice crystals, however, it was sufficient to obtain low-resolution information about the identity of the metals present in the asymmetric unit (a.u.).
